# Prognostic Impact of Percutaneous Coronary Intervention in Older Patients Hospitalized with Acute Myocardial Infarction: Real-World Findings from the Lombardy Health Database

**DOI:** 10.3390/jcm12175629

**Published:** 2023-08-29

**Authors:** Giancarlo Marenzi, Nicola Cosentino, Marta Resta, Claudia Lucci, Alice Bonomi, Filippo Trombara, Michele Della Rocca, Paolo Poggio, Olivia Leoni, Francesco Bortolan, Stefano Savonitto, Piergiuseppe Agostoni

**Affiliations:** 1 Centro Cardiologico Monzino, Istituti di Ricovero e Cura a Carattere Scientifico (IRCCS), 20138 Milan, Italy; nicola.cosentino@ccfm.it (N.C.); marta.resta@ccfm.it (M.R.); claudia.lucci@ccfm.it (C.L.); alice.bonomi@ccfm.it (A.B.); filippo.trombara@ccfm.it (F.T.); mic.dellarocca@gmail.com (M.D.R.); paolo.poggio@ccfm.it (P.P.); piergiuseppe.agostoni@ccfm.it (P.A.); 2 Regional Epidemiological Observatory, Lombardy Region, 20138 Milan, Italy; olivia_leoni@regione.lombardia.it (O.L.); francesco_bortolan@regione.lombardia.it (F.B.); 3 Manzoni Hospital, 23900 Lecco, Italy; s.savonitto@asst-lecco.it; 4 Cardiovascular Section, Department of Clinical Sciences and Community Health, University of Milan, 20138 Milan, Italy

**Keywords:** acute myocardial infarction, older patients, percutaneous coronary intervention, mortality, administrative database

## Abstract

Background. Older patients are less likely to receive percutaneous coronary intervention (PCI) for acute myocardial infarction (AMI) compared to younger patients. We investigated the prognostic impact of PCI in a large population of patients hospitalized with AMI in the period 2003–2018 by using the administrative Lombardy Health Database (Italy). Methods. We considered all patients aged ≥75 years hospitalized with AMI (either STEMI or NSTEMI) from 2003 to 2018 in Lombardy. Patients were grouped according to whether they were treated or not with PCI during the index hospitalization. The primary outcome was in-hospital mortality. The secondary endpoints were 1-year mortality and 1-year re-hospitalization for acute heart failure (AHF) or AMI. Results. 116,063 patients aged ≥75 years (mean age 83 ± 6; 48% males; 46% STEMI) were hospitalized with a primary diagnosis of AMI. Thirty-seven percent of them (*n* = 42,912) underwent PCI. The in-hospital mortality rate was significantly lower in PCI-treated patients (6% vs. 15%; *p* < 0.0001). One-year mortality and 1-year re-hospitalization for AHF/AMI were less frequent in PCI-treated patients (16% vs. 41% and 15% vs. 21%, respectively; *p* < 0.0001). The adjusted risks of the study endpoints were lower in PCI-treated patients: OR 0.37 (95% CI 0.36–0.39) for in-hospital mortality; HR 0.37 (95% CI 0.36–0.38) for 1-year mortality; HR 0.74 (95% CI 0.71–0.77) for 1-year re-hospitalization for AHF/AMI. Similar results were found in STEMI and NSTEMI patients considered separately. Conclusions. Our real-world data showed that in patients with AMI ≥ 75 years of age, PCI use is associated with lower in-hospital and 1-year mortality.

## 1. Introduction

The cornerstone of treatment of acute myocardial infarction (AMI) is percutaneous coronary intervention (PCI), as a primary revascularization strategy in patients with ST-segment elevation myocardial infarction (STEMI) and as an urgent invasive approach in those with non-ST-elevation myocardial infarction (NSTEMI) [1,2]. In both clinical settings, the increasing use of PCI has been associated with a substantial reduction in hospital and long-term mortality [1,2]. However, older patients, generally defined as those aged ≥75 years, are less likely to receive PCI for AMI compared to younger patients [3,4,5]. Historically, elderly patients were underenrolled in pivotal AMI trials, leading to a paucity of evidence regarding whether they benefit from PCI [1,2]. In addition, concerns about possible PCI-related side effects, in particular bleeding, renal, and vascular complications, multimorbidity, and medical futility, further complicate the AMI therapeutic decision-making process in this age group [1,2,6,7]. Thus, despite the high prevalence of elderly patients among those hospitalized with AMI [8,9], the increasing availability of PCI centers, and current guidelines stating that there is no upper age limit with respect to PCI indication in AMI [1,2], routine use of PCI for the treatment of older AMI patients still remains an unresolved issue.

In this study, we analyzed administrative data from Lombardy, the most populous Italian region with more than 10 million inhabitants, to evaluate the prognostic impact of PCI in patients aged ≥75 years hospitalized with AMI. Moreover, we investigated whether the rate of PCI use in elderly AMI patients has increased over the last 15 years and whether this was associated with improvements in overall hospital mortality, 1-year mortality, and 1-year re-hospitalization for acute heart failure (AHF) or AMI.

## 2. Methods

### 2.1. Data Source

The present study used linkable administrative health databases of the Lombardy region in Italy, which include a population registry with demographic data on all residents and detailed information on hospital records and drug prescriptions. Data are available for about 10 million registered inhabitants of Lombardy from 2000 to 2019. Access to data is allowed within the agreement between the Centro Cardiologico Monzino, I.R.C.C.S., Milan, Italy, and the Regional Health Ministry of Lombardy. Healthcare in Italy is publicly funded for all residents, irrespective of social class or employment, and everyone is assigned a personal identification number kept in the National Civil Registration System. All registered residents are assisted by general practitioners and are covered by the National Health System (NHS) with a high level of completeness regarding drug prescriptions, diagnosis, and length of observation. The pharmacy prescription database contains the medication name, anatomic therapeutic chemical classification code (ATC), and date of dispensation of drugs reimbursed by the NHS. The hospital database contains information on date of admission, discharge, death, primary diagnosis, and up to five co-existing clinical conditions and procedures performed. The diagnoses, uniformly coded according to the 9th International Code of Diseases (ICD-9-CM) and standardized for all Italian hospitals, are compiled by the hospital specialists directly in charge of the patients and validated by hospitals against detailed clinical-instrumental data as they determine reimbursement from the NHS. A unique identification code allows linkage of all databases. To ensure individual data protection, each identification code was automatically converted into an anonymous code before we received the dataset. In Italy, studies using retrospective anonymous data from administrative databases that do not involve direct access by investigators to identification data do not require Ethics Committee approval, notification, or patient informed consent.

### 2.2. Study Population

Patients with a hospitalization due to AMI (either STEMI or NSTEMI [ICD-9-CM codes 410.x]) from 1 January 2003 through 31 December 2018 were included in the analyses. Only hospitalizations in which an AMI-associated ICD-9 code was listed as a primary diagnosis were abstracted. When patients were transferred between hospitals, we evaluated the complete episode of care. Patients aged ≥75 years were grouped according to whether or not they underwent PCI during the index hospitalization. Patients undergoing urgent or elective coronary artery bypass grafting for the treatment of AMI were excluded from the study. Patients were then stratified into four groups according to the index hospitalization period: 2003–2006; 2007–2010; 2011–2014; and 2015–2018. Since medical data was recorded in the Lombardy registry in January 2000, past medical histories were available for all patients for at least 3 years before admission. Data collection was achieved by two trained reviewers.

### 2.3. Study Outcomes

The primary endpoint of the study was in-hospital mortality. One-year all-cause mortality and 1-year re-hospitalization for AHF or AMI were considered secondary endpoints. Patients were followed up from the index admission date until death, migration, or the end of one-year follow-up.

### 2.4. Statistical Analysis

Baseline characteristics were evaluated using descriptive statistics. Categorical variables were described using frequencies and percentages and compared using a Chi-square test; continuous variables were described using the mean and standard deviation (SD) and compared using a Student’s *t*-test. The differences between the patients stratified according to the four considered study periods were assessed by ANOVA and shown as a *p* for trend or Mantel-Haenszel χ^2^-test, as appropriate.

The association between in-hospital mortality and PCI was analyzed using a logistic model, and the results were reported as odds ratios (OR) and 95% confidence intervals (CI). The association between 1-year mortality and 1-year hospital readmission for AHF/AMI was investigated using either Cox regression or Cox regression to competing risk, as appropriate, and Kaplan-Meyer curves. The results were shown as the hazard ratio (HR) and 95% CI. The models were adjusted for all variables reported in the Table showing baseline characteristics of elderly patients treated or not treated with PCI and found to be significantly different between patients treated with and not treated with PCI. Medications taken before index hospitalization were considered for in-hospital mortality risk adjustment, while medications taken after hospital discharge were considered for 1-year endpoint risk adjustment.

We also performed propensity score matching. The score was used to match the following two cohorts: older patients treated with and not treated with PCI. The two groups were matched in a 1:1 ratio using all variables included in the Table showing baseline characteristics of elderly patients treated or not treated with PCI.

The preventable fraction of all endpoints associated with PCI was calculated as Pe × (1-OR [or HR]), where Pe is the prevalence of exposure and OR or HR is the risk for exposure relative to non-exposure.

All analyses were performed in the whole population, with STEMI and NSTEMI patients considered separately. A two-sided *p*-value less than 0.05 was required for statistical significance. All analyses were performed using SAS version 9.4 (SAS Institute, Cary, NC, USA).

## 3. Results

During the considered study period, 263,578 patients hospitalized with a primary diagnosis of AMI were identified. Of them, 116,063 (44%) patients were ≥75 years of age (mean age 83 ± 6 years; 52% females; 46% STEMI). The clinical characteristics of patients <75 years and ≥75 years are reported in Table 1.

Patients ≥ 75 years were less likely to undergo PCI during index hospitalization as compared to younger ones (37% vs. 66%; *p* < 0.0001). Older AMI patients had significantly higher in-hospital (12% vs. 3%; *p* < 0.0001) and 1-year (32% vs. 7%; *p* < 0.0001) mortality, as well as a higher rate of 1-year hospitalization for AHF/AMI (19% vs. 9%; *p* < 0.0001) than those <75 years of age.

### Older Patient Cohort

The clinical characteristics, chronic cardiovascular medications taken before index hospitalization and after discharge, and major in-hospital complications in older patients grouped according to PCI (yes vs. no) are shown in Table 2.

Patients treated with PCI were younger, more often STEMI patients, had fewer comorbidities, and experienced a less complicated in-hospital clinical course.

**Figure 1:**
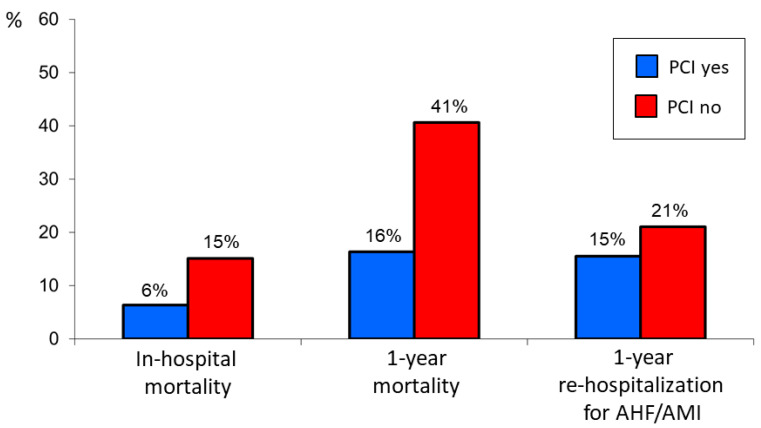
Primary and secondary endpoint rates in the overall older patient cohort treated or not treated with percutaneous coronary intervention (PCI). AHF = acute heart failure; AMI = acute myocardial infarction.

**Figure 2:**
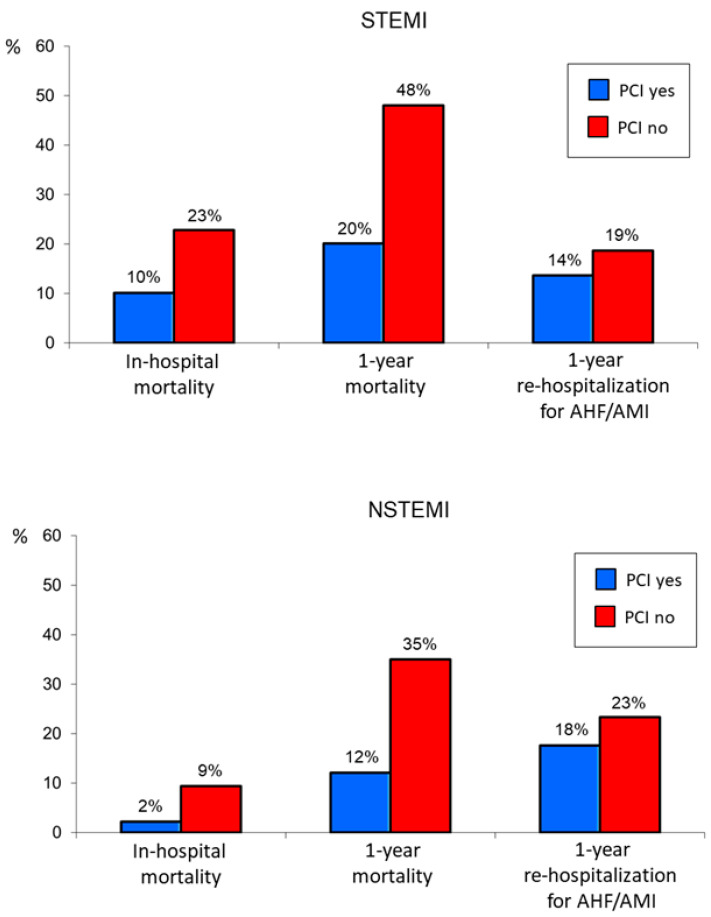
Primary and secondary endpoint rates in older STEMI (**upper** panel) and NSTEMI (**lower** panel) patients treated or not treated with percutaneous coronary intervention (PCI). AHF = acute heart failure; AMI = acute myocardial infarction.

The rates of the primary and secondary endpoints in the entire older population and in STEMI and NSTEMI patients considered separately were significantly lower in patients undergoing PCI as compared to those receiving medical therapy only (Figure 1 and Figure 2).

**Figure 3:**
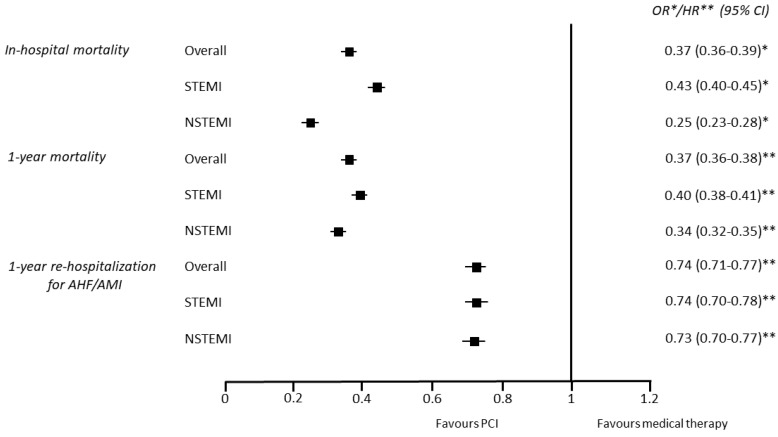
Adjusted risk of the primary and secondary endpoints associated with percutaneous coronary intervention (PCI) use in the overall cohort of patients ≥75 years and in STEMI and NSTEMI patients considered separately. Odds ratios and hazard ratios were adjusted for the variables reported in Table 2 and found to be significantly different between patients treated with and not treated with PCI, including medications taken before index hospitalization for OR and those taken after hospital discharge for HR. AHF = acute heart failure; AMI = acute myocardial infarction; CI = confidence interval; * OR = odds ratio; ** HR = hazard ratio.

Similarly, PCI use was associated with a lower adjusted risk of all the considered endpoints (Figure 3).

After propensity score matching, the study population included 79,222 older AMI patients (39,611 patients in each group). After matching, all variables were well balanced between the two groups. The risk of the primary and secondary endpoints was significantly lower in patients treated with PCI (Supplemental Material Figure S1).

**Figure 4:**
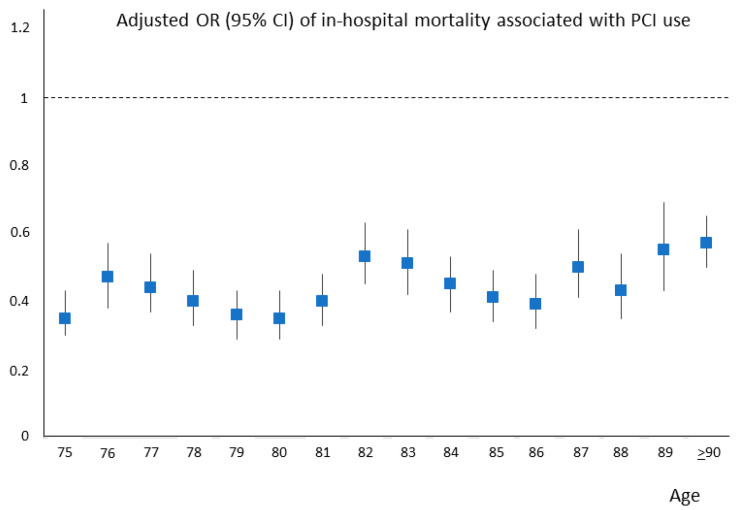
Adjusted risk of in-hospital mortality associated with percutaneous coronary intervention (PCI) use across ages. CI = confidence interval; OR = odds ratio.

The lower risk associated with PCI was maintained across the whole age spectrum in the entire AMI population (Figure 4), as well as in STEMI and NSTEMI patients considered separately (Supplemental Material Figure S2).

The Kaplan-Meyer curves for 1-year mortality and re-hospitalization for AHF/AMI in patients treated or not with PCI are shown in Supplemental Material Figure S3.

Among older patients not treated with PCI, 16,336 (22%) underwent diagnostic coronary angiography (Supplementary Table S1). Of them, 1277 (8%) underwent coronary artery bypass graft surgery during their index hospitalization. After adjustment for possible confounding factors, patients undergoing coronary angiography and treated conservatively experienced a significantly lower risk of in-hospital (OR 0.32 [95% CI 0.29–0.34]) and 1-year (HR 0.35 [95% CI 0.34–0.37]) mortality. Similarly, they had a lower risk of 1-year re-hospitalization for AHF/AMI (HR 0.77 [95% CI 0.73–0.81]).

The clinical characteristics of elderly patients stratified according to the four study periods are reported in Supplemental Material Table S2. The use of PCI progressively increased over time, from 22% during 2003–2006 to 49% during 2015–2018. In each time period, the rates and adjusted risks of in-hospital mortality and 1-year outcomes were lower in patients undergoing PCI than in those treated conservatively, without significant changes over time (Table 3).

**Figure 5:**
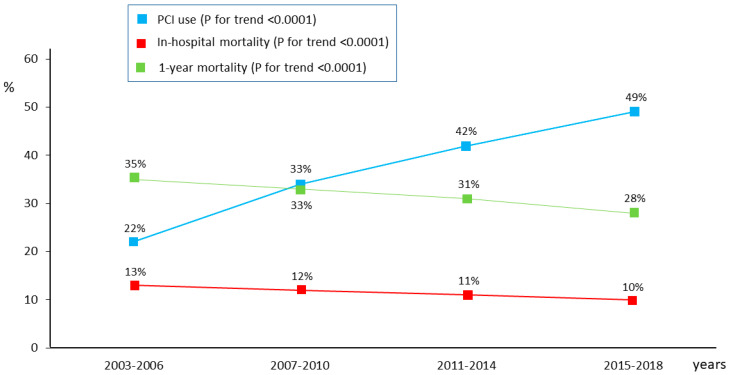
Temporal trend of in-hospital and 1-year mortality rates and percutaneous coronary intervention (PCI) use in the overall cohort of patients ≥75 years.

This was true both for the overall older population and for STEMI and NSTEMI patients considered separately. In parallel with the progressive increase in PCI use over time, overall in-hospital and 1-year mortality, as well as 1-year re-hospitalization for AHF/AMI, significantly decreased from 2003 to 2018 (Figure 5).

In the entire cohort of patients aged ≥75 years, the preventable fraction of in-hospital mortality associated with PCI (assuming that all patients were being treated with it) was 23% (26% in STEMI and 25% in NSTEMI patients). The preventable fractions of 1-year mortality and re-hospitalization for AHF/AMI associated with PCI were 26% (30% in STEMI and 25% in NSTEMI patients) and 12% (13% in STEMI and 10% in NSTEMI patients), respectively.

## 4. Discussion

Patients aged ≥ 75 years represent a large proportion of AMI hospitalizations, reaching 30% of those with STEMI and more than 40% of those with NSTEMI [10,11,12]. Despite significant advances in therapeutic strategies, the mortality rate of older patients with AMI is still high, with a 2- to 3-fold higher case fatality rate than that of younger patients [11,12,13]. To date, there is a paucity of systematic real-world data about the clinical outcomes when these patients are treated with PCI. This is mainly due to the underrepresentation or exclusion of elderly patients from major clinical trials of cardiovascular interventions, largely because of concerns about the increased risk of adverse events, complexity of follow-up, and limited life expectancy [1,2]. However, data from registries, hospital cohorts, and subgroup analyses from randomized trials have recently indicated beneficial effects on the outcome of PCI use irrespective of age in AMI patients [13,14,15,16,17,18]. As PCI remains underused in older patients, additional data are needed to confirm whether PCI is associated with a clinical benefit in this group of patients [3,4,5,13,14,15,16,17,18]. On these bases, we aimed at analyzing a large real-world administrative dataset to confirm the significant benefit described by meta-analyses of randomized trials comparing PCI with drug therapy in patients aged ≥ 75 years hospitalized with AMI [19,20].

In our study, we found that patients aged ≥ 75 years represented about 40% of all patients hospitalized with AMI in Lombardy between 2003 and 2018, with an overall in-hospital mortality rate of 12%, a 4-fold higher rate than that of patients younger than 75 years. Notably, in our cohort, PCI was performed in only 37% of older adults, compared to 66% of younger patients. However, when older patients underwent PCI, regardless of AMI type (STEMI or NSTEMI), their in-hospital mortality was significantly lower than that of AMI patients not treated with PCI. In particular, the adjusted risk of in-hospital mortality was 60% lower in PCI-treated patients. The lower mortality risk associated with PCI was maintained across increasing age, even in the very old. A similar behavior was observed when 1-year mortality and 1-year re-hospitalization for AHF/AMI were considered, with 60% and 25% lower adjusted risks, respectively, in patients treated with PCI. Again, this was true for both STEMI and NSTEMI patients. Thus, our findings are in line with the increasing evidence on the clinical efficacy of PCI in elderly AMI patients in a large real-world setting and further support the current recommendations of the European Society of Cardiology guidelines for the management of AMI patients. Indeed, they state that advanced age should not be considered an element of exclusion from primary PCI in STEMI (1) and that the same interventional strategy should be applied to older patients as to younger patients with NSTEMI [2]. Similar recommendations have been recently issued by a dedicated scientific statement of the American Heart Association [21].

To the best of our knowledge, ours is the largest study population focused on AMI patients older than 75 years, as almost all other studies included less than 3000–4000 patients [3,4,5,11,12,13,14,15,16,17,18,19,20,22]. Only one administrative Spanish study can be compared to ours [16]. Indeed, it considered 107,890 elderly AMI patients and reported a favorable impact of PCI on the in-hospital mortality rate. Yet, differently from our study, the authors included STEMI patients only and limited their observation to the hospital phase.

In the present study, we also evaluated whether PCI use changed over the considered time frame of 15 years and whether this was associated with a change in hospital and 1-year outcomes. Although the mortality risk difference associated with PCI in our cohort of older AMI patients remained unchanged over time, in-hospital mortality and 1-year outcomes progressively decreased across years. As PCI use increased from 22% during 2003–2006 to almost 50% during 2015–2018, it can be speculated that increased PCI use contributed, at least in part, to the gradual reduction in overall AMI mortality over the study period. Of note, this trend was also true when STEMI and NSTEMI patients were considered separately.

Although there are several aspects that remain to be investigated in our study, including PCI-related complications, our data strongly support the European Society of Cardiology guidelines [1,2] that advanced age per se should not be considered a decisive factor in precluding PCI in patients hospitalized with AMI.

Therefore, future studies should not focus on defining an age limit beyond which PCI would not be indicated but, rather, on determining for which older patients PCI should be considered a futile treatment. Moreover, in this study, among patients not treated with PCI, those undergoing diagnostic coronary angiography experienced lower in-hospital and 1-year mortality than those not undergoing coronary angiography. This suggests that coronary angiography during AMI hospitalization, regardless of PCI use, is a useful prognostic stratification tool in older patients, allowing for the identification of the more appropriate therapeutic strategy (medical therapy versus surgical or percutaneous myocardial revascularization).

Administrative databases are a reliable tool to describe the outcomes of large cohorts representing the real clinical care setting since they collect data over time in a standardized fashion and, by the way, at a low cost. However, limitations that are typical of all the studies based on administrative datasets need to be acknowledged. Administrative data can suffer from systematic biases as their quality depends on the accuracy of coding. In particular, analyses relied on accurate coding of AMI and other conditions of interest, and biases may have resulted from underreporting or changes in diagnosis or coding patterns over time. Yet, it should be highlighted that the endpoints considered in the present study, in particular in-hospital and 1-year mortality, are less likely to be affected by coding errors. Second, some specific pieces of information on clinical variables or laboratory tests closely associated with AMI prognosis, in particular left ventricular ejection fraction, renal function, extent of coronary artery disease, completeness of myocardial revascularization, and late presentation in STEMI patients, were not available [23,24]. Similarly, with respect to the old patient, key variables of functional status, cognitive status, patient preferences, and hospitalization ward that could influence PCI referral were not available. Thus, the reasons why conservatively treated patients did not undergo PCI cannot be completely inferred from our data. Finally, the generalizability of our findings to other countries may be limited.

## 5. Conclusions

In conclusion, the present study based on large real-world data showed that in patients ≥ 75 years of age hospitalized with AMI, PCI use is associated with significantly lower in-hospital and 1-year mortality, as well as a reduction in 1-year re-hospitalization for AHF/AMI, compared to conservative treatment. The progressive increase in PCI use over the years may partially account for the improved outcome across years in older AMI patients.

## Figures and Tables

**Table 1:** Baseline characteristics of the overall study population and of patients grouped according to age.

	Overall Study Population (*n* = 263,564)	Age < 75 Years (*n* = 147,501)	Age ≥ 75 Years (*n* = 116,063)	*p* Value
**Age** (years)	70 ± 13	61 ± 9	83 ± 6	<0.001
**Biological Sex** (female)	93,363 (35%)	32,983 (22%)	60,360 (52%)	<0.001
STEMI, *n* (%)	138,939 (53%)	85,543 (58%)	53,396 (46%)	<0.001
NSTEMI, *n* (%)	124,639 (47%)	61,972 (42%)	62,667 (54%)	<0.001
**History of comorbidities** (in the previous 2 years), *n* (%)				
Cerebrovascular disease	7428 (3%)	2237 (1%)	5191 (5%)	<0.001
Hypertension	82,728 (31%)	36,253 (25%)	46,475 (40%)	<0.001
Diabetes mellitus	66,029 (25%)	31,632 (21%)	34,397 (30%)	<0.001
Chronic IHD	58,023 (22%)	30,836 (21%)	27,187 (23%)	<0.001
Prior AMI	26,593 (10%)	12,278 (8%)	14,315 (12%)	<0.001
Atrial fibrillation	15,910 (6%)	4184 (3%)	11,726 (10%)	<0.001
Chronic renal disease	17,005 (6%)	5982 (4%)	11,023 (9%)	<0.001
COPD	14,044 (5%)	4371 (3%)	9673 (8%)	<0.001
Cancer	24,188 (9%)	10,780 (7%)	13,408 (12%)	<0.001
**Number of comorbidities** *n* (%)				
0	104,326 (39%)	70,2784 (48%)	34,042 (29%)	<0.001
1	82,180 (31%)	44,809 (30%)	37,371 (32%)	
2	46,574 (18%)	21,228 (14%)	25,346 (22%)	
3	20,414 (7%)	7909 (5%)	12,515 (11%)	
>3	10,060 (4%)	3271 (2%)	6789 (6%)	
**Medications of interest** (before index AMI), *n* (%)				
ACEi/ARB	137,794 (52%)	62,788 (42%)	75,006 (65%)	<0.001
Beta blockers	84,169 (32%)	40,460 (27%)	43,709 (38%)	<0.001
Diuretics	62,755 (24%)	17,598 (12%)	45,157 (39%)	<0.001
Calcium-antagonists	75,656 (29%)	30,778 (21%)	44,878 (39%)	<0.001
Lipid lowering drugs	91,012 (35%)	49,443 (33%)	41,569 (36%)	<0.001
Antiplatelet drugs	116,252 (44%)	51,693 (35%)	64,559 (56%)	<0.001
Oral anticoagulant drugs	14,974 (6%)	4630 (3%)	10,344 (9%)	<0.001
Anti-hyperglycemic drugs	57,968 (22%)	27,456 (18%)	30,512 (26%)	<0.001

Abbreviations: ACEi = angiotensin-converting enzyme inhibitors; AMI = acute myocardial infarction; ARB = angiotensin receptor blockers; COPD = chronic obstructive pulmonary disease: IHD = ischemic heart disease; NSTEMI = non-ST-elevation myocardial infarction; STEMI = ST-elevation myocardial infarction.

**Table 2:** Baseline characteristics of elderly patients treated or not treated with percutaneous coronary intervention.

	PCI Yes (*n* = 42,912)	PCI No (*n* = 73,151)	*p* Value
**Age** (years)	81 ± 4	84 ± 6	<0.001
**Age groups** (years)			<0.001
75–80	22,124 (52%)	22,853 (31%)	
81–85	13,550 (32%)	21,554 (29%)	
86–90	5865 (14%)	17,746 (24%)	
>90	1373 (3%)	10,998 (15%)	
**Biological Sex** (female)	18,801 (44%)	41,579 (57%)	<0.001
STEMI, *n* (%)	22,297 (52%)	31,099 (43%)	<0.001
NSTEMI, *n* (%)	20,615 (48%)	42,052 (57%)	<0.001
**History of comorbidities** (in the previous 2 years), *n* (%)			
Cerebrovascular disease	1032 (2%)	4159 (6%)	<0.001
Hypertension	17,303 (40%)	29,172 (40%)	0.134
Diabetes mellitus	12,167 (28%)	22,230 (30%)	<0.001
Chronic IHD	10,468 (24%)	16,719 (23%)	<0.001
Prior AMI	3921 (9%)	10,394 (14%)	<0.001
Atrial fibrillation	3175 (7%)	8551 (12%)	<0.001
Chronic renal disease	2792 (7%)	8231 (11%)	<0.001
COPD	2281 (5%)	7392 (10%)	<0.001
Cancer	5005 (12%)	8403 (11%)	0.36
**Number of comorbidities** *n* (%)			
0	12,976 (30%)	21,066 (29%)	<0.001
1	14,624 (34%)	22,747 (31%)	
2	9377 (22%)	15,969 (22%)	
3	4167 (10%)	8348 (11%)	
>3	1768 (4%)	5021 (7%)	
**Medications of interest** (before index AMI), *n* (%)			
ACEi/ARB	27,253 (64%)	47,753 (65%)	<0.001
Beta blockers	16,739 (39%)	26,970 (37%)	<0.001
Diuretics	12,293 (29%)	32,864 (45%)	<0.001
Calcium-antagonists	16,168 (38%)	28,710 (39%)	<0.001
Lipid lowering drugs	17,100 (40%)	24,469 (33%)	<0.001
Antiplatelet drugs	22,837 (53%)	41,722 (57%)	<0.001
Oral anticoagulant drugs	3307 (8%)	7037 (10%)	<0.001
Anti-hyperglycemic drugs	10,898 (25%)	19,614 (27%)	<0.001
**Major in-hospital complications, *n* (%)**			
Cardiogenic shock, *n* (%)	2189 (5%)	4353 (6%)	<0.0001
AKI, *n* (%)	926 (2%)	2315 (3%)	<0.0001
Atrial fibrillation, *n* (%)	4830 (11%)	12,294 (17%)	<0.0001
Acute heart failure, *n* (%)	6733 (16%)	24,469 (33%)	<0.0001
Mechanical ventilation, *n* (%)	1172 (3%)	1524 (2%)	0.43

Abbreviations: ACEi = angiotensin-converting enzyme inhibitors; AKI = acute kidney injury requiring renal replacement therapy; AMI = acute myocardial infarction; ARB = angiotensin receptor blockers; COPD = chronic obstructive pulmonary disease: IHD = ischemic heart disease; NSTEMI = non-ST-elevation myocardial infarction; STEMI = ST-elevation myocardial infarction.

**Table 3:** Primary and secondary endpoint rates and risks in the four study periods in elderly patients treated or not treated with percutaneous coronary intervention.

2003–2006	2007–2010	2011–2014	2015–2018	*p* for Trend	
**Overall**	*n* = 26,951	*n* = 30,237	*n* = 30,251	*n* = 28,624	
PCI, *n* (%)	5953 (22%)	10,177 (34%)	12,336 (42%)	14,146 (49%)	<0.0001
**In-hospital mortality**, *n* (%)					
Overall	3550 (13%)	23,829 (13%)	3400 (11%)	2971 (10%)	<0.0001
PCI yes	385 (6%)	664 (6%)	804 (6%)	871 (6%)	0.2613
PCI no	3165 (15%)	23,165 (16%)	2596 (15%)	2100 (14%)	0.0347
OR (95% CI)	0.39 (0.34–0.44)	0.37 (0.34–0.41)	0.39 (0.36–0.43)	0.39 (0.35–0.42)	
**1-year mortality**, *n* (%)					
Overall	9336 (35%)	10,013 (33%)	9268 (31%)	8046 (28%)	<0.0001
PCI yes	913 (15%)	1658 (16%)	2090 (16%)	2325 (16%)	0.095
PCI no	8423 (40%)	8355 (42%)	7178 (41%)	5721 (39%)	0.221
HR (95% CI)	0.33 (0.31–0.35)	0.31 (0.31–0.35)	0.35 (0.33–0.36)	0.36 (0.34–0.3)	
**1-year re-hospitalization for AHF/AMI**, *n* (%)					
Overall	6309 (23%)	6607 (22%)	5326 (18%)	4022 (14%)	<0.0001
PCI yes	1129 (19%)	1915 (19%)	1918 (15%)	1702 (12%)	<0.0001
PCI no	5180 (25%)	4692 (23%)	3408 (16%)	2320 (16%)	<0.0001
HR (95% CI)	0.49 (0.46–0.53)	0.51 (0.49–0.54)	0.53 (0.51–0.56)	0.53 (0.50–0.56)	
**STEMI**	*n* = 15,313	*n* = 14,872	*n* = 12,632	*n* = 10,579	
PCI, *n* (%)	3822 (25%)	5674 (38%)	6327 (50%)	6474 (61%)	<0.0001
**In-hospital mortality**, *n* (%)					
Overall	2668 (17%)	2657 (18%)	2177 (17%)	1837 (17%)	0.61
PCI yes	338 (9%)	570 (10%)	643 (10%)	710 (11%)	0.001
PCI no	2330 (20%)	2087 (23%)	1534 (24%)	1127 (27%)	<0.001
OR (95% CI)	0.38 (0.34–0.43)	0.38 (0.34–0.42)	0.35 (0.32–0.39)	0.32 (0.29–0.36)	
**1-year mortality**, *n* (%)					
Overall	5804 (38%)	5611 (38%)	4479 (35%)	3533 (33%)	<0.0001
PCI yes	677 (18%)	1140 (20%)	1298 (21%)	1373 (21%)	<0.0001
PCI no	5127 (45%)	4471 (49%)	3181 (50%)	2160 (53%)	<0.0001
HR (95% CI)	0.33 (0.31–0.36)	0.34 (0.32–0.36)	0.33 (0.31–0.35)	0.32 (0.30–0.34)	
**1-year re-hospitalization for AHF/AMI**, *n* (%)					
Overall	3169 (21%)	2719 (18%)	1814 (14%)	1138 (11%)	<0.0001
PCI yes	672 (18%)	927 (16%)	808 (13%)	634 (10%)	<0.0001
PCI no	2497 (22%)	1792 (19%)	1006 (16%)	504 (12%)	<0.0001
HR (95% CI)	0.49 (0.45–0.53)	0.50 (0.47–0.54)	0.47 (0.44–0.50)	0.52 (0.48–0.56)	
**NSTEMI**	*n* = 11,638	*n* = 15,365	*n* = 17,619	*n* = 18,045	
PCI, *n* (%)	2131 (18%)	4503 (29%)	6309 (36%)	7672 (46%)	<0.0001
**In-hospital mortality**, *n* (%)					
Overall	882 (8%)	1172 (8%)	1223 (7%)	1134 (6%)	<0.0001
PCI yes	47 (2%)	94 (2%)	161 (3%)	161 (2%)	0.91
PCI no	835 (9%)	1.078 (10%)	1062 (9%)	973 (9%)	0.39
OR (95% CI)	0.23 (0.17–0.31)	0.19 (0.16–0.24)	0.25 (0.21–0.30)	0.21 (0.17–0.24)	
**1-year mortality**, *n* (%)					
Overall	3532 (30%)	4402 (29%)	4789 (27%)	4513 (25%)	<0.0001
PCI yes	236 (11%)	518 (11%)	792 (13%)	952 (12%)	0.04
PCI no	3296 (35%)	3884 (36%)	3997 (35%)	3561 (34%)	0.45
HR (95% CI)	0.27 (0.24–0.31)	0.27 (0.25–0.30)	0.30 (0.28–0.33)	0.31 (0.29–0.33)	
**1-year re-hospitalization for AHF/AMI**, *n* (%)					
Overall	3140 (27%)	3888 (25%)	3512 (20%)	2884 (16%)	<0.0001
PCI yes	457 (21%)	988 (22%)	1110 (18%)	1068 (14%)	<0.0001
PCI no	2863 (28%)	2900 (27%)	2402 (21%)	1816 (18%)	<0.0001
HR (95% CI)	0.45 (0.40–0.51)	0.50 (0.46–0.54)	0.53 (0.49–0.57)	0.43 (0.40–0.47)	

AHF = acute heart failure; HR = Hazard ratio (PCI yes vs. PCI no); AMI = acute myocardial infarction; NSTEMI = non-ST-elevation myocardial infarction; OR= Odds ratio (PCI yes vs. PCI no), PCI = percutaneous coronary intervention; STEMI = ST-elevation myocardial infarction.

## Data Availability

Data subject to third party restrictions.

## Supplementary Materials

The following supporting information can be downloaded at: https://www.mdpi.com/article/10.3390/jcm12175629/s1, Table S1. Baseline characteristics of the older patients not treated with percutaneous coronary intervention, grouped according to coronary angiography performance. Table S2. Baseline characteristics of older patient cohort hospitalized with acute myocardial infarction, stratified according to the four study periods. Figure S1. Risk of the primary and secondary endpoints associated with percutaneous coronary intervention (PCI) use in the matched cohort of patients >75 years and in STEMI and NSTEMI patients considered separately. AHF = acute heart failure; AMI = acute myocardial infarction; CI = confidence interval; HR = hazard ratio; OR = odds ratio. Figure S2. Adjusted risk of 1-year mortality (upper panel) and 1-year re-hospitalization for acute heart failure (AHF) or acute myocardial infarction (AMI) (lower panel) associated with percutaneous coronary intervention (PCI) use across ages. CI = confidence interval; HR = hazard ratio. Figure S3. Kaplan-Meier curves for secondary endpoints stratified by percutaneous coronary intervention (PCI) use in the overall older patient cohort. AHF = acute heart failure; AMI = acute myocardial infarction.
