# Supplementary material for: Prognostic Impact of Percutaneous Coronary Intervention in Older Patients Hospitalized with Acute Myocardial Infarction: Real-World Findings from the Lombardy Health Database
Source: J Clin Med. 2023 Aug 29;12(17):5629. doi: 10.3390/jcm12175629 (PMC10488530; doi:10.3390/jcm12175629)
Supplement: Supplementary file 1 [file jcm-12-05629-s001.zip › jcm-2526497-supplementary.pdf]

Supplementary Table 1. Baseline characteristics of the older patients not treated with percutaneous coronary intervention, grouped according to coronary angiography performance.

	**Coronary Angiography yes** (n=16,336)	**Coronary Angiography no** (n=56,815)	**P value**
**Age** (years)	81±4	85±6	<0.001
**Gender** (female)	8,009 (49%)	33,570 (59%)	<0.001
STEMI, n (%)	5,030 (31%)	26,069 (46%)	<0.001
NSTEMI, n (%)	11,306 (69%)	30,746 (54%)	<0.001
**History of comorbidities** (in the previous 2 years), n (%)			
Cerebrovascular disease	530 (3%)	3,629 (6%)	<0.001
Hypertension	7,080 (43%)	22,092 (39%)	<0.001
Diabetes mellitus	4,815 (29%)	17,415 (31%)	0.0039
Chronic IHD	3,656 (22%)	13,063 (23%)	0.10
Prior AMI	1,715 (10%)	8,679 (15%)	<0.0001
Atrial fibrillation	1,578 (10%)	6,973 (12%)	<0.001
Chronic renal disease	1,129 (7%)	7,102 (12%)	<0.001
COPD	1,097 (7%)	6,295 (11%)	<0.001
Cancer	1,797 (11%)	6,606 (12%)	0.02
**Number of comorbidities** n (%)			<0.001
0	4,621 (28%)	16,445 (30%)	
1	5,573 (34%)	17,174 (30%)	
2	3,707 (23%)	12,262 (22%)	
3	1,655 (10%)	6,693 (12%)	
>3	780 (5%)	4,241 (7%)	
**Medications of interest** (before index AMI), n (%)			
ACEi/ARB	11,164 (68%)	36,589 (64%)	<0.001
Beta blockers	6,752 (41%)	20,218 (36%)	<0.001
Diuretics	5,594 (34%)	27,270 (48%)	<0.001
Calcium-antagonists	6,430 (39%)	22,280 (39%)	0.73
Lipid lowering drugs	7,114 (44%)	17,355 (31%)	<0.001
Antiplatelet drugs	9,165 (56%)	32,557 (57%)	0.006
Oral anticoagulant drugs	1,577 (10%)	5,460 (10%)	0.86
Anti-hyperglycemic drugs	4,360 (27%)	15,254 (27%)	0.68
**Medications of interest** (after index hospital discharge)*, n (%)			
ACEi/ARB	11,164 (68%)	36,589 (64%)	<0.0001
Beta blockers	6,752 (41%)	20,218 (36%)	<0.0001
Diuretics	5,594 (34%)	27,270 (48%)	<0.0001
Calcium-antagonists	6,430 (39%)	22,280 (39%)	0.73

Lipid lowering drugs	7,114 (43%)	17,355 (30%)	<0.0001
Antiplatelet drugs	9,165 (56%)	32,557 (57%)	0.006
Oral anticoagulant drugs	1,577 (10%)	5,460 (10%)	0.86
Anti-hyperglycemic drugs	4,360 (27%)	15,254 (27%)	0.68

Abbreviations: ACEi=angiotensin-converting enzyme inhibitors; AMI=acute myocardial infarction; ARB=angiotensin receptor blockers; COPD=chronic obstructive pulmonary disease: IHD=ischemic heart disease; NSTEMI=non-ST-elevation myocardial infarction; PCI=percutaneous coronary intervention; STEMI=ST-elevation myocardial infarction.

*Medications after hospital discharge were computed in patients surviving index hospitalization.

Supplementary Table 2. Baseline characteristics of older patient cohort hospitalized with acute myocardial infarction, stratified according to the four study periods.

	**Pts hospitalized between 2003-2006** (n=26,951)	**Pts hospitalized between 2007-2010** (n=30,237)	**Pts hospitalized Between 2011-2014** (n=30,251)	**Pts hospitalized between 2015-2018** (n=28,624)	**P for trend**
**Age** (years)	83±6	83±5	82±6	83±6	<0.001
**Age groups** (years), n (%)					<0.001
75-80	11,225 (42%)	11,790 (10%)	11,215 (10%)	10,747 (9%)	
81-85	8,406 (31%)	9,386 (8%)	9,023 (8%)	8,289 (7%)	
86-90	4,413 (16%)	6,216 (5%)	6,760 (6%)	6,222 (5%)	
>90	2,907 (11%)	2,845 (2%)	3,253 (3%)	3,366 (3%)	
**Gender** (females), n (%)	14,726 (55%)	16,292 (54%)	15,484 (51%)	13,878 (48%)	
STEMI	15,313 (57%)	14,872 (49%)	12,632 (42%)	10,579 (37%)	<0.001
NSTEMI	11,638 (43%)	15,365 (51%)	17,619 (58%)	18,045 (63%)	<0.001
PCI during index hospitalization, n (%)	5,953 (22%)	10,177 (34%)	12,636 (42%)	14,145 (49%)	<0.001
**History of comorbidities** (in the previous 2 years), n (%)					
Cerebrovascular disease	1,702 (6%)	1,499 (5%)	1,158 (4%)	832 (3%)	<0.001
Hypertension	9,903 (37%)	12,040 (40%	12,271 (42%)	12,261 (43%)	<0,001
Diabetes mellitus	7,248 (27%)	8,822 (29%)	9,224 (30%)	9,103 (32%)	<0.001
Chronic IHD	5319 (20%)	6,211 (21%)	7,294 (24%)	8,363 (29%)	<0.001
Prior AMI	3,716 (14%)	4,133 (14%)	3,615 (12%)	2,851 (10%)	<0.001
Atrial fibrillation	2,681 (10%)	2,987 (10%)	3,075 (10%)	2,983 (10%)	0.03
Chronic renal disease	2,475 (9%)	3,035 (10%)	2,743 (9%)	2,770 (10%)	0.60
COPD	2,760 (10%)	2,830 (9%)	2,191 (7%)	1,892 (7%)	<0.001
Cancer	2,688 (10%)	3,323 (11%)	3,534 (12%)	3,863 (13%)	<0.001
**Number of comorbidities** n (%)					
0	9,250 (34%)	9,350 (31%)	8,521 (28%)	6,921 (24%)	<0.001

1	8,230 (31%)	9,639 (32%)	10,054 (33%)	9,448 (33%)	
2	5,291 (20%)	6,294 (21%)	6,756 (22%)	7,005 (24%)	
3	2,676 (10%)	3,119 (10%)	3,189 (11%)	3,531 (12%)	
>3	1,504 (6%)	1,835 (6%)	1,731 (6%)	1,719 (6%)	
**Medications of interest** (before index hospitalization), n (%)					
ACE-I/ARBS	16,218 (60%)	19,573 (65%)	20,289 (67%)	18,926 (66%)	<0.001
Beta blockers	20,044 (74%)	19,730 (66%)	17,538 (58%)	15,042 (53%)	<0.001
Diuretics	10,800 (40%)	11,941 (34%)	11,741 (39%)	10,675 (37%)	<0.001
Ca-antagonists	11,136 (41%)	11,900 (39%)	11,556 (38%)	10,286 (36%)	<0.001
Lipid lowering drugs	6,364 (24%)	9,765 (32%)	12,287 (41%)	13,153 (46%)	<0.001
Antiplatelet drugs	13,955 (52%)	17,424 (58%)	18,336 (61%)	14,844 (52%)	0.036
Oral anticoagulant drugs	1,681 (6%)	2,419 (8%)	2,853 (9%)	3,391 (12%)	<0.001
Antihyperglycemic drugs	6,385 (24%)	7,840 (26%)	8,186 (27%)	8,101 (28%)	<0.001
**Medications of interest** (after index hospital discharge)*, n (%)					
ACE-I/ARBS	2,954 (13%)	3,070 (12%)	2,448 (9%)	1,481 (6%)	0.002
Beta blockers	2,100 (9%)	2,605 (10%)	2,485 (9%)	1,754 (7%)	<0.0001
Diuretics	3,661 (16%)	3,845 (15%)	3,316 (12%)	2,136 (8%)	<0.0001
Ca-antagonists	1,103 (5%)	1,290 (5%)	1,035 (4%)	781 (3%)	<0.0001
Lipid lowering drugs	1,746 (7%)	2,283 (9%)	2,178 (8%)	1,547 (6%)	<0.0001
Antiplatelet drugs	2,813 (12%)	3,107 (12%)	2,722 (10%)	1,713 (7%)	<0.0001
Oral anticoagulant drugs	690 (3%)	830 (3%)	766 (3%)	692 (3%)	<0.0001
Antihyperglycemic drugs	1,224 (5%)	1,459 (6%)	1,296 (5%)	811 (3%)	<0.0001

Abbreviations: ACEi=angiotensin-converting enzyme inhibitors; AMI=acute myocardial infarction; ARB=angiotensin receptor blockers; COPD=chronic obstructive pulmonary disease: IHD=ischemic heart disease; NSTEMI=non-ST-elevation myocardial infarction; PCI=percutaneous coronary intervention; STEMI=ST-elevation myocardial infarction.

*Medications after hospital discharge were computed in patients surviving index hospitalization.

Supplemental Material Figure 1.

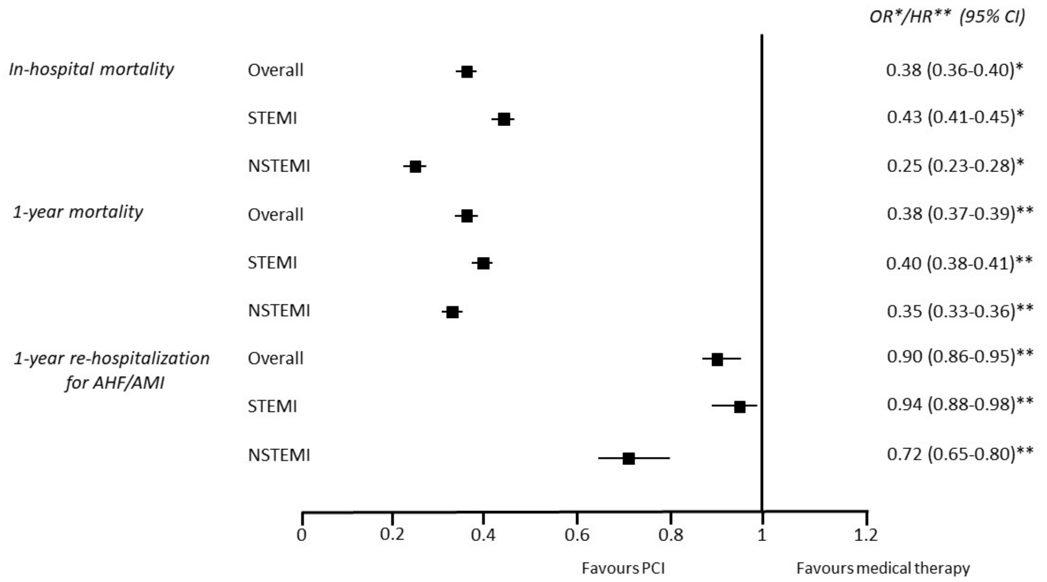

Supplemental Material Figure 1.

Risk of the primary and secondary endpoints associated with percutaneous coronary intervention (PCI) use in the matched cohort of patients ≥75 years and in STEMI and NSTEMI patients considered separately. AHF = acute heart failure; AMI = acute myocardial infarction; CI = confidence interval; HR = hazard ratio; OR = odds ratio.

Supplemental Material Figure 2.

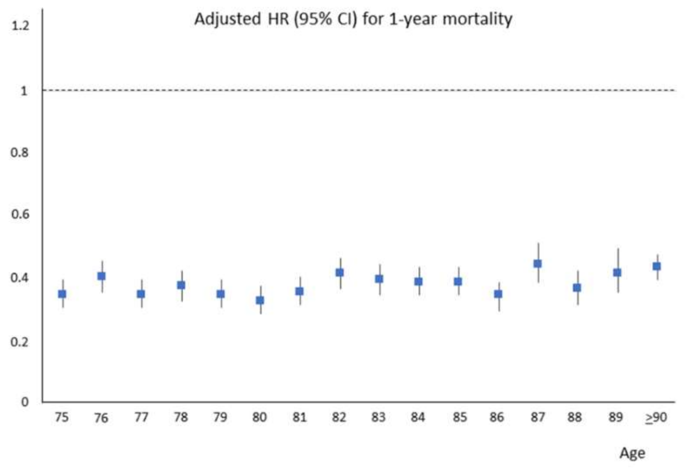

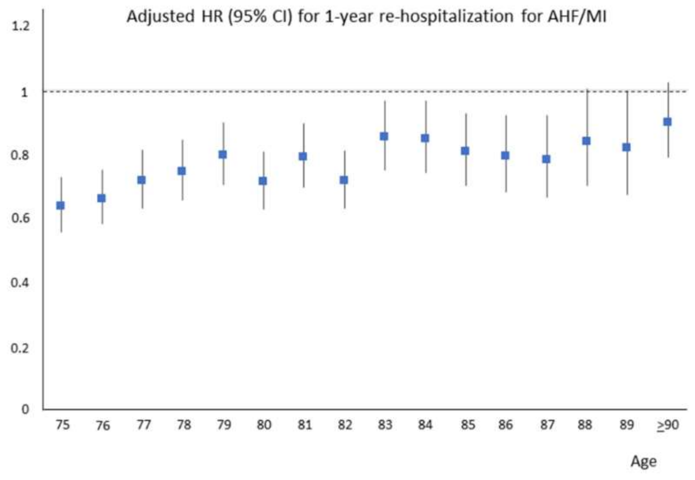

Supplemental Material Figure 2. Adjusted risk of 1-year mortality (upper panel) and 1-year re-hospitalization for acute heart failure (AHF) or acute myocardial infarction (AMI) (lower panel) associated with percutaneous coronary intervention (PCI) use across ages. CI = confidence interval; HR = hazard ratio.

Supplemental Material Figure 3.

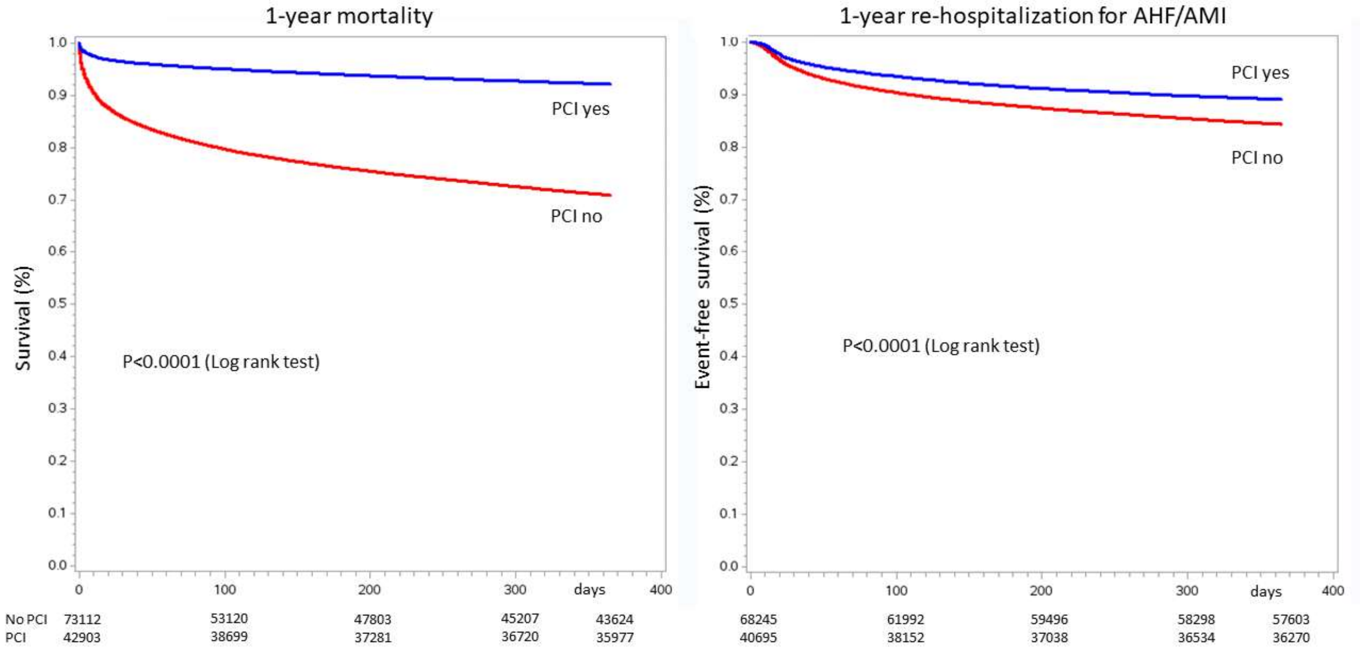

Supplemental Material Figure 3. Kaplan-Meier curves for secondary endpoints stratified by percutaneous coronary intervention (PCI) use in the overall older patient cohort. AHF = acute heart failure; AMI = acute myocardial infarction.
